# Light Field Reconstruction Using Residual Networks on Raw Images

**DOI:** 10.3390/s22051956

**Published:** 2022-03-02

**Authors:** Ahmed Salem, Hatem Ibrahem, Hyun-Soo Kang

**Affiliations:** 1 School of Information and Communication Engineering, College of Electrical and Computer Engineering, Chungbuk National University, Cheongju 28644, Korea; ahmeddiefy@chungbuk.ac.kr (A.S.); hatem@chungbuk.ac.kr (H.I.); 2 Electrical Engineering Department, Faculty of Engineering, Assiut University, Assiut 71515, Egypt

**Keywords:** light field, view synthesis, angular super-resolution, convolutional neural network

## Abstract

Although Light-Field (LF) technology attracts attention due to its large number of applications, especially with the introduction of consumer LF cameras and its frequent use, reconstructing densely sampled LF images represents a great challenge to the use and development of LF technology. Our paper proposes a learning-based method to reconstruct densely sampled LF images from a sparse set of input images. We trained our model with raw LF images rather than using multiple images of the same scene. Raw LF can represent the two-dimensional array of images captured in a single image. Therefore, it enables the network to understand and model the relationship between different images of the same scene well and thus restore more texture details and provide better quality. Using raw images has transformed the task from image reconstruction into image-to-image translation. The feature of small-baseline LF was used to define the images to be reconstructed using the nearest input view to initialize input images. Our network was trained end-to-end to minimize the sum of absolute errors between the reconstructed and ground-truth images. Experimental results on three challenging real-world datasets demonstrate the high performance of our proposed method and its outperformance over the state-of-the-art methods.

## 1. Introduction

**Figure 1:**
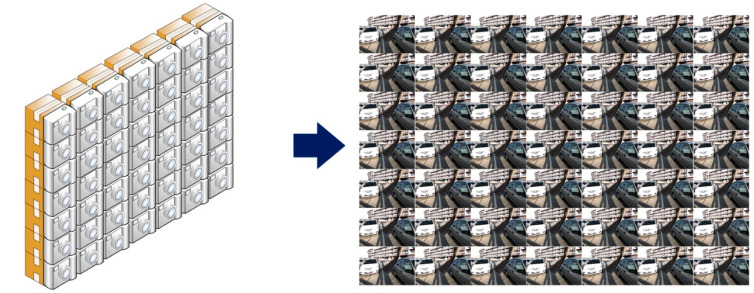
LF imaging captures arrays of light from all directions separately, unlike conventional imaging, which captures intensity from only one direction. This can be imagined as a group of cameras taking pictures of the same scene at the same moment. These cameras are arranged as shown and placed very close to each other so that adjacent images differ slightly from each other. This massive data capture opens completely new possibilities. For example, specific parts of the image can be changed to be blurry or in focus, 3D images can be created from only one exposure, and even the depth of field can be changed.

Light-Field (LF) photography is characterized by its ability to provide more information about 3D space compared to conventional photography methods, as shown in Figure 1. LF records arrays of light coming from all directions separately, unlike conventional photography which captures only the 2D projection of the perceived light by integrating light rays [1,2,3,4]. LF photography has gained more attention with the emergence of commercial LF cameras and the huge number of applications, such as saliency detection [5], depth-sensing [6], de-occlusion [7], post-capture refocusing [8], object segmentation [9], light-field stitching [10], light-field coding [11], light-field quality assessment [12], and so on.

Before the introduction of commercial LF cameras, camera arrays [3] and computer-controlled gantry [13] were used to capture LF, which were disadvantaged by being bulky and expensive. The introduction of commercial LF cameras has provided a portable and cheap solution and solved the aforementioned problems by placing a micro-lens array ahead of the image sensor to encode the angular information of the upcoming rays [14,15]. Regrettably, densely sampled LF cannot be obtained in both spatial and angular domains owing to the limited sensor resolution of commercial LF cameras.

In order to tackle the limited-resolution problem and obtain high-resolution (HR) densely sampled LF images, many studies have been conducted to super-resolve LF images [16,17,18,19,20,21], and other studies have been conducted to reconstruct densely sampled LF from a sparse set of views [22,23,24,25,26,27,28,29,30,31,32,33,34,35,36,37]. LF image super-resolution is beyond the scope of this research, as we focus on LF reconstruction, or LF angular super-resolution in other words.

Intending to reconstruct LF from a sparse set of views, some researchers build their work by inferring depth based on the consistency between input views and then warp input views based on the inferred depth to reconstruct densely sampled LF [22,23]. However, depth inferring and view warping are complex processes, especially for LF images with small disparity, which makes it easy to introduce errors and not maintain image consistency. Other learning-based methods have been proposed to reconstruct densely sampled LF without explicit depth estimation [25,29]. These methods were designed to upsample Epipolar Plane Images (EPIs) in one or more directions. However, such a method cannot explore sufficiently the angular relationships and hence cannot restore more texture details. Of the methods which worked on raw LF images (lenslet images), Gul and Gunturk [38] proposed a shallow network to enhance both spatial and angular resolution. Their network consists of two smaller networks. The first doubles the angular resolution, and the second doubles the spatial resolution of LF images.

In an attempt to mitigate the aforementioned problems and to improve the quality of LF reconstruction, this paper proposes a deep residual network to reconstruct densely sampled LF images with a small baseline from a small set of input views. Our model was trained end-to-end using raw LF images. The raw LF image is generated by encoding the captured 2D array of images into a single image. Using raw images for training allows the network to understand and model the relationship between different images of the same scene and hence provide better quality. To generate the input raw image, we took advantage of the small-baseline feature and initialized the required views using nearest-view initialization.

Several experiments were conducted on challenging datasets to show the capability of our model to reconstruct LF images with high quality. The main contributions of our paper are as follows:

- We take advantage of the features of small-baseline LF and use the nearest-view method to initialize the views to be reconstructed using the nearest input view. In addition, using raw LF images eases our task by transforming it from an image reconstruction into an image-to-image translation.
- We propose a deep Residual Convolutional Neural Network (CNN) to work on raw LF images to reconstruct high-quality LF images.

## 2. Related Work

Owing to the limited sensor resolution of commercial LF cameras, densely sampled LF cannot be obtained in both spatial and angular domains. LF angular reconstruction has been studied by many researchers to tackle the inherent trade-off between spatial and angular resolutions. The research in this area can be categorized into two groups, regarding whether the model is depth-dependent or depth-independent.

### 2.1. Depth-Dependent LF Reconstruction

Depth-dependent methods divide the learning process into two stages: depth estimation and LF estimation. In the first stage, a depth map is predicted for each view being reconstructed, and the estimated depth is used to warp the input views to reconstruct new views. In the second stage, the warped images go through an optimization stage as the estimated depth is usually inaccurate and noisy. These two stages are trained end-to-end to reduce the reconstruction error. Many traditional methods were proposed to follow this scheme. For example, a variational framework was proposed by Wanner and Goldluecke [39]. In their framework, they estimated depth maps locally using EPI analysis. Then, these depth maps were improved and used to reconstruct LF images using convex optimization algorithms. Later on, they improved their work by reformulating the problem as a continuous inverse problem, which allowed foreshortening effects to be taken into account [40]. Mitra and Veeraraghavan provided a common framework for some LF tasks using a patch-based approach where they modeled LF patches using a Gaussian mixture model (GMM) [41]. Pendu et al. [42] proposed using a regularized least square regression to obtain depth layers for scene representation. These layers can be shifted and filtered to reconstruct views at different positions.

Among the learning-based approaches to reconstruct a densely sampled LF, Kalantari et al. [22] model this pipeline using two consecutive networks to first estimate disparity and then predict color. These networks were trained end-to-end to reconstruct views at arbitrary positions with high quality. As this method reconstructs each view separately, it could not learn the relationship between different images, nor could it provide a good reconstruction quality at occluded regions. Different from Kalantari’s method, which proposed to reconstruct LF images with a small baseline, Jin et al. [23] proposed to reconstruct LF images with a large baseline using depth information. This method proposes to calculate a depth map for each image to be created, then, after using this depth to warp all input images, the warped images are blended to reconstruct the final views. In the blending stage, spatial and angular dimensions are alternatively processed by convolutional layers to explore the directional relations between different images, which is the same as the technique used in [17].

### 2.2. Depth-Independent LF Reconstruction

Depth-independent methods implicitly learn depth information to reconstruct densely sampled LF images. Of the traditional methods based on signal processing, Shi et al. [43] proposed a reconstruction method that improves the sparsity in the continuous Fourier spectrum. This method is based on their observation that the LF spectrum is much sparser in the continuous domain than in the discrete domain. They used this method to improve reconstruction quality and reduce sampling requirements. Vagharshakyan et al. [44] developed a rendering technique utilizing the EPI representation in the Shearlet domain along with an iterative regularization algorithm. This method showed great performance for scenes with semi-transparent objects.

With the great success achieved by deep learning, many learning-based approaches have been proposed to solve this problem. For example, Yoon et al. [28,36] developed a method to upsample LF in both spatial and angular resolutions. However, this method was limited to one reconstruction task (3 × 3 views from 2 × 2 views). During the reconstruction process, and except for the central view, the new views are reconstructed using neighboring views in the horizontal or vertical direction only. Such limited use of the angular information affected the reconstruction quality. Yeung et al. [37] used 2D alternating spatial–angular convolutions to reconstruct LF for several tasks. However, the model produces false shadows and ghosting artifacts at the boundaries of reconstructed views due to the ignoring of the relations between different views.

Since EPIs can reflect consistency, some learning-based methods have been developed in the EPI domain. Wu et al. [29] reconstructed LF using low-frequency components of the LF instead of direct restoration, to reduce the ghosting artifacts. A blur kernel was selected to extract the low-frequency components, where the restoration process is modeled by a CNN. Finally, a deblur kernel is used to recover high-frequency components. Later on, they indicated that the sheared EPI has a clear structure when the depth and sheared value are equal, where they trained another CNN to learn fusion scores for upsampled EPIs with different shearing values [32,33]. However, they underused the angular information by utilizing EPIs in one direction only. Wang et al. [30] developed a model to reconstruct LF using EPI and EPI stacks, where 2D and 3D CNN were utilized to build a pseudo-4D CNN. Later on, they improved the reconstruction quality by applying EPI structure-preserving loss [34]. However, they used the angular information insufficiently by utilizing only horizontal or vertical EPI stacks for the reconstruction. In addition, they upsampled LF in a hierarchical way, which means more error accumulation on the last reconstructed views. Liu et al. [25] proposed a multi-angular epipolar-based network using horizontal, vertical, and two angular EPI stacks for the reconstruction process. However, while they used rich angular information better than the previous methods, it was not enough, as they used only one EPI stack in each direction.

## 3. Methodology

### 3.1. Problem Formulation

**Figure 2:**
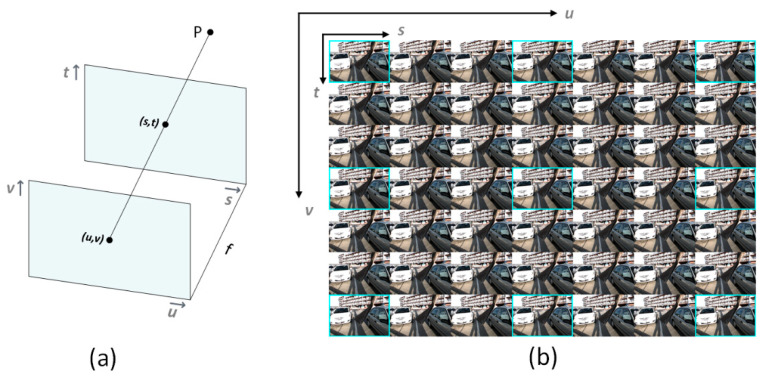
Four-dimensional LF parameterization and visualization. (**a**) Two-plane parameterization of LF where the incident ray from a 3D point space intersects with the first plane at angular position (*u*,*v*) and with the second plane at spatial position (*s*,*t*). (**b**) The 4D LF can be visualized as a 2D array of Sub-Aperture Images (SAI). In our method, 3 × 3 views bordered in light blue are used to reconstruct 7 × 7 views.

The most common way to parameterize the 4D LF is the two-plane parameterization, where ray lines are defined by their intersections with two planes at any position [2]. The coordinate system on the first and second planes are (*u*,*v*) and (*s*,*t*), respectively, as shown in Figure 2. Therefore, from any point P in a 3D space, the light ray that intersects the two planes at angular position (*u*,*v*) and spatial position (*s*,*t*) can be represented by *L*(*u*,*v*,*s*,*t*). The 4D LF can be visualized as a 2D array of Sub-Aperture Images (SAI), where the same scene is shown by neighboring images with a small disparity.

Given a sparse set of LF images $LF^{\prime }\in \;R^{u\times v\times s\times t}$, (*u*,*v*) represents the angular coordinate or number of input views, and (*s*,*t*) represents the spatial coordinate or spatial resolution of each input view. Our goal is to reconstruct a dense set of LF images $LF\in \;R^{U\times V\times s\times t}$ with *U* × *V* views, where *U* > *u* and *V* > *v*. In our method, (*U* × *V* = 7 × 7) views are reconstructed from (*u* × *v* = 3 × 3) views. Finally, 40 novel views are reconstructed to increase the angular resolution from 3 × 3 to 7 × 7.

### 3.2. Raw LF Image Reconstruction

**Figure 3:**
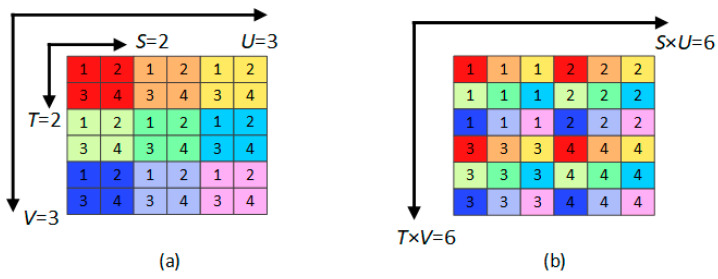
Raw LF image reconstruction: (**a**) A simple case of 3 × 3 views of LF where each view is represented by one color and contains 4 pixels numbered 1–4, (**b**) The mapping from the LF array of views with size (*U*,*V*,*S*,*T*) into raw LF image of size (*U* × *S*, *V* × *T*).

In our method, for the sake of utilizing the angular information along with the spatial information sufficiently, we propose to construct raw LF images and use them to train our network. A Periodic Shuffling Operator (PS) is used to rearrange the 2D array of LF images $\in R^{U\times V\times s\times t}$ into one single image $\in R^{Us\times Vt}$, as proposed in [45]. In our method, 7 × 7 views with spatial resolution H and W are to be reconstructed. Therefore, the input and output from the network will be a raw LF $\in R^{7H\times 7W},\;$ while the 2D array of the same LF images $\in R^{7\times 7\times H\times W}$. A simple case of mapping from the 2D arry representation into the raw LF is shown in Figure 3.

### 3.3. Nearest-View Initialization Method

**Figure 4:**
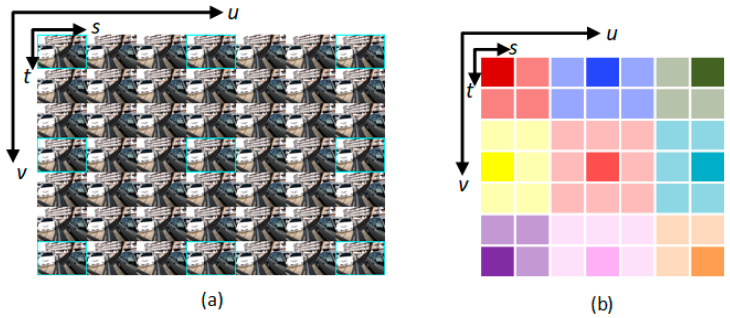
Nearest-view initialization method. (**a**) A 2D array of LF images where 7 × 7 views are to be reconstructed from 3 × 3 views bordered in light blue. (**b**) Input views (shown in bright colors) are used to assign their close neighboring views (shown in light colors) with the same value. After this initialization for all the views to be reconstructed, the raw LF images are created to be used as an input to the network.

Using raw LF images eases our task by transforming it from an image reconstruction into an image-to-image translation task. However, the input raw images need to be initialized to the same size as output images. One of the characteristics of LF images with a small baseline is that adjacent images have a small disparity between them. Accordingly, we used this fact to initialize the views to be reconstructed with the nearest input view, as shown in Figure 4. To the best of our knowledge, the nearest-view initialization method has not been used before. However, this method has a limitation, where only the views which are connected to the input view can be initialized with this input view (a view is to be connected to the input view if their edges or corners touch).

Our assumption is based on the geometric analysis of LF rendering presented by Lin and Shum [46], where they investigated the problem of minimum sampling for anti-aliased LF rendering and they proved that depth variation of the scene and camera-resolution determine sampling rate. They concluded that to render a view from two successive views, the sample spacing between these views must not exceed one pixel for an estimate of exact depth while it is two pixels with the optimal constant depth.

Our model will learn depth information implicitly like other depth-independent models as mentioned in [26]. As long as depth information is available, the allowable sample spacing between two successive views can be increased up to two pixels, which is the case in the LF with a small baseline. Instead of rendering from two successive views, we are rendering from just one view to initialize the raw LF input-image.

### 3.4. Network Architecture

#### 3.4.1. Overview

**Figure 5:**
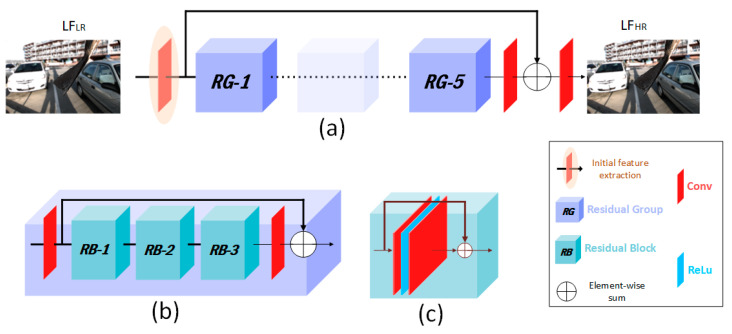
Overview of the proposed network architecture, which reconstructs densely sampled LF through mapping from low-resolution into high-resolution raw LF images. (**a**) After raw LF reconstruction, initial features are extracted to be fed to the network to extract and restore deeper features. As shown, the network contains five Residual Groups (RG) with a skip connection. (**b**) Each RG contains three Residual Blocks (RB) with another skip connection. (**c**) RB is the main unit of our network, which consists of cascaded convolutions and rectified linear units with a skip connection.

Our network is similar to the one proposed in [47]. The proposed network can be divided into two parts in terms of functionality, as shown in Figure 5: Initial feature extraction, and deep feature extraction and restoration. The proposed network was trained only on the luminance component while the chrominance components were calculated using the nearest-view initialization method as explained before. Our network was trained to learn mapping from the Low-Resolution (LR) raw LF image $LF_{LR}$ to the High-Resolution (HR) LF image $LF_{HR}$. The problem can be written as: $LF\prime_{HR}=f\left(LF_{LR},\;\theta \right)$.

In the calculation, f(.) represents the function that maps from LR image to HR image, and θ represents the network parameters to be learned through training.

The input raw LF image denoted as $LF_{LR}$ is first reconstructed from the sparse input views and then fed to the network. Initial features are then extracted from the input images to be fed to the main part of the network. For this part, we used one Convolutional layer (Conv). As proposed in [47], the extracted features are then fed to a deep Residual-In-Residual (RIR) structure, where the original RIR structure contains 10 Residual Groups (RG) and a skip connection. Each RG contains 20 Residual Blocks (RB). We used only five RGs and three RBs in our implementation as the use of more RGs and RBs did not contribute significantly to quality improvement but slowed down our network due to increased computations. In addition, they proposed a channel attention mechanism inside each RB to exploit more contextual information. However, such a mechanism does not suit the nature of raw LF images. Our RB is identical to the one used in [48], which consists of two convolutional layers with a Rectified Linear Unit (ReLU) in between. Similar to Single-Image Super-Resolution (SISR) approaches [47,48,49], the goal of skip connections is to bypass low-frequency information to let the network concentrate more on the high-frequency information.

#### 3.4.2. Loss Function

Various loss functions are utilized to optimize CNN training, such as *L*_2_ (sum of all the squared differences), and *L*_1_ (sum of all the absolute differences). As mentioned by Zhao et al. [50], In terms of measuring tools used to evaluate the quality of images such as PSNR and SSIM, the *L*_1_ loss is reported to achieve improved performance compared to the *L*_2_ loss.

The proposed network was trained to reduce the *L*_1_ distance between the raw *LF* input image and its corresponding ground-truth image. Given a training set containing *N* pairs of input and ground-truth images, the *L*_1_ loss is defined as:

$$L_{1}\left(\theta \right)=\frac{1}{N}\overset{N}{\underset{i=1}{\sum }}{\left|LF_{HR}^{i}-f\left(LF_{LR}^{i}\right)\right|}_{1}$$

where f(.) represents the function that maps from *LR* input image $LF_{LR}$ to *HR* image $LF\prime_{HR}$, and θ represents the network parameters to be learned through the training.

#### 3.4.3. Training Details

We used 100 LF images for training from the publicly available datasets [22,51]. These images were captured using Lytro Illum cameras with 14 × 14 angular resolution, and 376 × 541 spatial resolution. To escape from optical distortion and light falloff, we took the middle 7 × 7 views for training. First, we mapped the 2D array of LF images from size (*u* = 7, *v* = 7, *s* = 541, *t* = 376) to reconstruct the raw LF image of size (*u* × *s* = 3787, *v* × *t* = 2632). We extracted patches of size 128 × 128 with a stride of one from input and ground-truth images to prepare the training dataset. Our training dataset contained 58,000 pairs of input and corresponding ground-truth patches which were sufficient for training, and 16 LF patches of luminance component were randomly extracted in every training batch. Our model was trained by ADAM optimizer [52] with β1 = 0.9, β2 = 0.999, and ǫ = 10^−8^. The initial learning rate was set to 2 × 10^−4^ and then decreased exponentially by a rate of 0.1 every 100 epochs. Our model was trained for 120 epochs in TensorFlow [53] with NVIDIA GeForce RTX 3090 GPU.

## 4. Experiments and Discussion

To show the capability of our model in reconstructing LF images with high quality, we compare it with state-of-the-art learning-based models for LF reconstruction, including Wu et al. [29], Wu et al. [33], Yeung et al. [37], Liu et al. [25], and Zhang et al. [26]. Three real-world datasets were used for the comparisons, named 30 Scenes [22], Refractive & Reflective surfaces, and Occlusions from Stanford Lytro Light Field Archive [51]. To measure the LF reconstruction quality, Average PSNR and SSIM are used for the luminance images over all the reconstructed views.

### 4.1. Comparison with the State-of-the-Art

Numerical results in terms of (PSNR/SSIM) are presented in Table 1. It is clear from the results that the proposed model achieves the best reconstruction quality with 0.61 dB, 1.3 dB, 1.56 dB, 2.27 dB, and 4.13 dB average PSNR increases over Zhang et al. [26], Liu et al. [25], Yeung et al. [37], Wu et al. [29], and Wu et al. [33], respectively. In addition, our model achieves the best SSIM on two datasets and the second-best result on one dataset by a very small margin. Wu et al. [29] underused the angular information by utilizing EPIs in one direction only. Later on, they produced better results by utilizing EPIs in horizontal and vertical directions. However, they hierarchically upsampled LF, which means more error accumulation on the last reconstructed views [33]. Yeung et al.’s [37] model produces false shadows and ghosting artifacts at the boundaries of reconstructed views due to the ignoring of the relations between different views. Liu et al. [25] used angular information better than the previous methods; however, it was not enough, as they used only one EPI stack in each direction. Zhang et al. [26] used micro-lens images and view image stacks to explore more LF information. However, we attribute the significant improvement in the results of our model to the initialization of the input image, where the network is fine-tuning rather than learning. In addition, using the raw LF images enables the network to understand and model the relationship between different images of the same scene well and thus restore more texture details and provide better quality.

**Figure 6:**
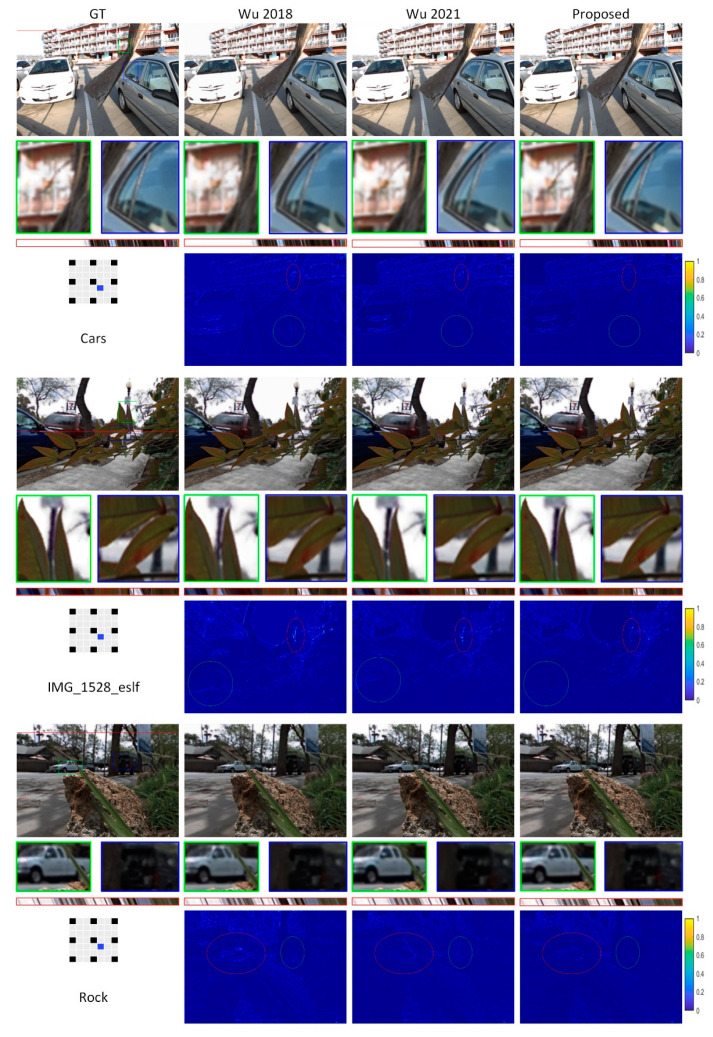
Visual comparison of LF image reconstruction with other methods together with the corresponding ground-truth images. Error maps between reconstructed luminance images and corresponding ground-truth are also shown. On the left side of the error maps, black images represent input views, gray the images to be reconstructed, and blue the displayed view. Extracted EPIs are shown in red boxes in addition to a close-up of image portions in blue and green boxes. Error maps demonstrate the capability of our method. For example, in the Cars scene, the green circle indicates the error around the rear tire of the car.

A visual comparison between the reconstructed views of three real-world scenes by our model and two other models is shown in Figure 6. Even with occluded regions and complex backgrounds, our model can reconstruct better-quality views with clear edges around object boundaries. Error maps provide a better comparison between the reconstructed images. For example, in the IMG_1528_eslf scene, the red ellipse shows the error on the complex region between the two leaves of the tree, where the distance is small and the lamppost is occluded in some input views.

### 4.2. Ablation Study

We compare four different architectures to show the effect of different components of the model as shown in Table 2. All the models shown in Table 2 have the same number of convolutional layers but with different residual connections, where RG, RB, and f indicate the number of Residual Groups, the number of Residual Blocks, and the number of filters for each convolutional layer, respectively. The goal of residual (skip) connections is to bypass low-frequency information to let the network concentrate more on the high-frequency information. This effect is clearly shown by the results achieved from the first model without RGs and RBs and the last model with RGs and RBs even with the same number of convolutional layers.

After that, we compare seven different models with a varying number of Residual Groups and Residual Blocks as shown in Table 3. As shown, SSIM value is slightly affected by changing the number of other variables. This indicates that working on raw LF images preserves the perceptual quality of the reconstructed LF images even with small models such as the one in the first raw. By the increase in the number of RGs and RBs used, the model provides better performance, until some points when the model starts overfitting. Although there is a better model than the proposed one, the difference between them is very small. In addition, by increasing the size of the model, the time required increases due to the increase in complexity.

## 5. Future Work

The proposed method for LF reconstruction on raw images shows impressive results even with complex scenes. However, our method is limited to reconstruction tasks where the number of views to be created is only two views between the input scenes. This is due to the limitation of the nearest-view initialization technique where only the views which are connected to the input view can be initialized with this input view. For example, our method would fail to produce good results when reconstructing 8 × 8 views out of 2 × 2 views. In the future, we plan to develop an initialization technique for other reconstruction tasks to map the problem from image reconstruction into image-to-image translation.

## 6. Conclusions

We have proposed a learning-based LF reconstruction method. To effectively explore the non-local property of 4D LF, we adopted raw LF representation, which enabled the network to understand and model the relationship well and thus restore more texture details and provide better quality. We initialized the views to be reconstructed using the nearest-view method, along with the raw LF representation, and the task was transformed from image reconstruction into image-to-image translation. Our method improves the average PSNR over the second-best method by 0.64 dB.

## Figures and Tables

**Table 1:** Numerical comparison (PSNR/SSIM) of the proposed model with the state-of-the-art models to reconstruct 7 × 7 views out of 3 × 3 views on datasets 30 Scenes, Reflective, and Occlusions.

Dataset	Wu [29]	Wu [33]	Yeung [37]	Liu [25]	Zhang [26]	Proposed
30 Scenes	41.40/0.980	43.592/0.986	44.66/0.990	44.86/0.991	45.68/0.992	45.96/0.991
Reflective	42.19/0.974	43.092/0.977	43.90/0.9793	44.31/0.980	44.92/0.982	45.41/0.984
Occlusions	37.25/0.925	39.748/0.948	40.00/0.953	40.16/0.957	40.80/0.955	41.86/0.962
Average	40.28/0.959	42.14/0.971	42.85/0.974	43.11/0.976	43.80/0.976	44.41/0.979

**Table 2:** Numerical comparison (PSNR/SSIM) of four different models with the same number of convolutional layers but with different residual connections. The results of the proposed model are in bold.

RG	RB	30 Scenes	Reflective	Occlusions	Average
X	X	37.21/0.956	40.55/0.969	34.60/0.938	37.45/0.955
X	√	45.64/0.990	45.22/0.982	41.52/0.960	44.13/0.978
√	X	45.89/0.991	45.42/0.983	41.78/0.961	44.36/0.978
√	√	**45.96/0.991**	**45.41/0.984**	**41.86/0.962**	**44.41/0.979**

**Table 3:** Numerical comparison (PSNR/SSIM) of seven different models with a varying number of Residual Groups and Residual Blocks to reconstruct 7 × 7 views out of 3 × 3 views on dataset 30 Scenes, Reflective, and Occlusions. The results of the proposed model are in bold.

	30 Scenes	Reflective	Occlusions	Average
RG = 3, RB = 3, f = 32	44.97/0.991	44.68/0.982	40.84/0.962	43.50/0.978
RG = 3, RB = 3, f = 64	45.54/0.990	45.11/0.981	41.41/0.960	44.02/0.977
RG = 4, RB = 3, f = 64	45.83/0.992	45.32/0.984	41.71/0.960	44.28/0.979
RG = 6, RB = 3, f = 64	45.82/0.992	45.30/0.985	41.65/0.962	44.25/0.980
RG = 5, RB = 2, f = 64	45.82/0.992	45.33/0.984	41.75/0.963	44.30/0.980
**RG = 5, RB = 3, f = 64**	**45.96/0.991**	**45.41/0.984**	**41.86/0.962**	**44.41/0.979**
RG = 5, RB = 4, f = 64	46.02/0.993	45.49/0.985	41.94/0.963	44.48/0.980

## Data Availability

The datasets used in this paper are public datasets. We also provide the test and the evaluation codes of the proposed method at: https://github.com/ahmeddiefy/LF_Raw which was created (accessed on 28 January 2022).
